# Insect and plant invasions follow two waves of globalisation

**DOI:** 10.1111/ele.13863

**Published:** 2021-08-22

**Authors:** Aymeric Bonnamour, Jérôme M. W. Gippet, Cleo Bertelsmeier

**Affiliations:** ^1^ Department of Ecology and Evolution University of Lausanne Lausanne Switzerland

**Keywords:** biological invasions, globalisation waves, insects, invasion rate, sampling effort, temporal dynamics, trade openness, vascular plants

## Abstract

Globalisation has facilitated the spread of alien species, and some of them have significant impacts on biodiversity and human societies. It is commonly thought that biological invasions have accelerated continuously over the last centuries, following increasing global trade. However, the world experienced two distinct waves of globalisation (~1820–1914, 1960‐present), and it remains unclear whether these two waves have influenced invasion dynamics of many species. To test this, we built a statistical model that accounted for temporal variations in sampling effort. We found that insect and plant invasion rates did not continuously increase over the past centuries but greatly fluctuated following the two globalisation waves. Our findings challenge the idea of a continuous acceleration of alien species introductions and highlight the association between temporal variations in trade openness and biological invasion dynamics. More generally, this emphasises the urgency of better understanding the subtleties of socio‐economic drivers to improve predictions of future invasions.

## Graphical abstract

It is commonly thought that biological invasions have accelerated continuously over the last centuries, following increasing global trade. However, the world experienced two distinct waves of globalization (~1820–1914, 1960‐present), and it remains unclear whether these two waves have influenced invasion dynamics of many species. To test this, we built a statistical model that accounted for temporal variations in sampling effort, and found that insect and plant invasion rates did not continuously increase over the past centuries, but greatly fluctuated following the two globalization waves.

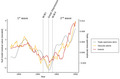

## INTRODUCTION

Since the Industrial Revolution, increasing global trade and human movement have facilitated the spread of thousands of species worldwide (Chapman et al., 2017; Early et al., 2016; Hulme, 2009)⁠. Some of these species, the so‐called alien species, have managed to survive and reproduce outside of their native range without human assistance. Alien species can reduce local biodiversity and impair ecosystem functioning (Bellard et al., 2016; Cameron et al., 2016; Castro‐Díez et al., 2019; Pyšek & Richardson, 2010)⁠, contribute to the homogenisation of floras and faunas (Capinha et al., 2015; Winter et al., 2009)⁠, cause agricultural losses (Paini et al., 2016)⁠ and affect human health and well‐being (Bacher et al., 2018; Pyšek & Richardson, 2010)⁠. Over the past two centuries, development of new trade technologies and infrastructures has influenced the spread dynamics of alien species (Hulme, 2021)⁠. Trade and transport are likely to be important drivers of future biological invasions (Essl et al., 2020; Lenzner et al., 2020) and global species flows from donor to recipient regions might change following shifts in trade dynamics (Epanchin‐Niell et al., 2021)⁠. Understanding how variations in global trade have influenced the spread of alien species in the past is, therefore, crucial to better predict invasion risk and prevent alien species introductions in the future.

It has been suggested that biological invasions have accelerated over the last centuries, driven by increasing global trade (Seebens et al., 2017)⁠. However, although globalisation of trade and transport has intensified dramatically since the Industrial Revolution, this increase was not continuous. Instead, the world experienced two major waves of globalisation (Baldwin & Martin, 1999; Federico & Tena‐Junguito, 2017)⁠. It is a great challenge for invasion biology to understand how these two waves have affected invasion dynamics. So far, the two waves of globalisation have gone largely unnoticed in ecology, yet the economic literature recognises these waves as the dominant feature of global commerce during the last two centuries (Baldwin & Martin, 1999). The first globalisation wave occurred from ~1820 to 1914, powered by the decline in international transport costs due to the development of steamships and railroad network (Baldwin & Martin, 1999; Federico & Tena‐Junguito, 2017; O’Rourke & Williamson, 2002),⁠ which led to an unprecedented rise in world trade. The first wave of globalisation ended with the outbreak of the First World War and the protectionist reactions to the Great Depression, which strongly reduced international trade (Baldwin & Martin, 1999; Federico & Tena‐Junguito, 2017)⁠⁠. The second wave of globalisation started after the Second World War, around 1960, as international trade increased almost continuously until the early 21st century, driven among other factors by the development of transportation networks (Baldwin & Martin, 1999; Federico & Tena‐Junguito, 2017)⁠⁠.

These important variations in the intensity of global exchanges are likely to have influenced biological invasions over the past 200 years—as suggested by a previous study on ants (Bertelsmeier et al., 2017). Yet, previous research on other taxa does not seem to confirm this. Indeed, for most taxa, the rate of alien species first records (i.e. the annual number of alien species first records per country) was relatively low in the 19th century and increased continuously until the end of the 20th century (Seebens et al., 2017)⁠. However, scientific activities recording species occurrences have also strongly increased over the past two centuries, and a variety of sources of species record data have emerged (Boakes et al., 2010)⁠. Consequently, alien species first record rates are likely to be strongly influenced by the global increase in species observations, which could mask underlying dynamics of biological invasions. Accounting for variations in sampling effort is, therefore, required to disentangle the invasion dynamics from sampling bias (Belmaker et al., 2009; Costello & Solow, 2003; Mangiante et al., 2018; Solow & Costello, 2004) and, thus, get a more precise understanding of the temporal dynamics of invasions and of the potential impact of the two waves of globalisation.

To address these questions, we performed a global temporal analysis of alien species first record rates, which accounted for variations in sampling effort over time. As a proxy for sampling effort, we used the first record rate of native species, sourced from the Global Biodiversity Information Facility (GBIF; https://www.gbif.org). GBIF aims at gathering all the information about species taxonomy and distribution worldwide from various sources not only museums, local and global databases, scientific publications but also geotagged smartphone photos from amateur naturalists in the more recent years (Heberling et al., 2021). It offers a global coverage of more than 1.6 billion occurrences for about 1.6 million species spanning several centuries. This makes GBIF data appropriate to account for temporal variations in sampling effort worldwide.

We used these data to build a null model of alien species first record rate to (i) estimate biological invasion dynamics after accounting for variations in sampling effort and (ii) test the link between invasions dynamics and temporal variations in trade openness as a measure of trade globalisation. To do that, we focused on insects and vascular plants as they are the taxa with the highest numbers of first records (7,918 and 16,348 alien species first records, respectively), and they cover the majority of biodiversity and alien species described so far (Scheffers et al., 2012; Seebens et al., 2017). Moreover, these two groups include many species transported and introduced unintentionally (Saul et al., 2017)⁠ and are, therefore, likely to be influenced by variations in international trade.

## MATERIAL AND METHODS

We used a null model that assumes that the rate of alien species introduction is constant over time and simulates the rate of first records per country using sampling effort as a predictor. To account for variations in sampling effort, we used the rate of native species first records per country as a proxy. To measure the link between sampling effort and alien species first records, we computed the correlation between the first record rate simulated with the null model and the observed first record rate. Then, we computed the null model residuals (i.e. difference between the simulated and observed alien species first records), which represent variations in first record rate unexplained by sampling effort, and thus reflect true biological invasion dynamics. Finally, we measured the correlation between the residuals and variations in world trade openness to test whether the spread dynamics of alien insects and vascular plants followed the two waves of globalisation. Our approach to analyse the residuals of a model with a single predictor to remove first the confounding effect of this predictor is commonly used in the literature (Brown et al., 2021; Sofaer & Jarnevich, 2017)⁠.

To confirm our results, we also used a second approach where we directly included trade openness as predictor variable in the model by defining the introduction rate as a function of world trade openness (described in more detail in the Supplementary Material S1). Moreover, we tested the effect of other economic indexes (world trade and GDP) in addition to trade openness as a measure of globalisation to assess which one better explained biological invasions dynamics when sampling bias is accounted for (Supplementary Material S1).

### Alien species first record data

We extracted data for insects and vascular plants from the Alien Species First Records database (Seebens et al., 2017)⁠, which is a global data set of alien species first record dates per country for a wide range of taxonomic groups. It contains data from different sources including online databases, scientific publications, books and personal collections. As part of the Alien Species First Records database was not published online, we excluded from our analysis countries for which data were missing. For vascular plants, we excluded the United States, New Zealand, Germany, Italy and Mexico from our analysis. For insects, only Australia was excluded. We removed first records with the ‘casual’ status (i.e. records of non‐established alien species) to consider only established alien species (i.e. species that established permanent self‐sustaining populations). Finally, the analysis was restricted to first records between 1750 and 2000. We did not include data after 2000 as they are incomplete due to lags in the recording of new alien species (Seebens et al., 2017)⁠. This resulted in a total of 7,918 first records of 4,528 established alien insect species, and 16,348 first records of 6,030 established alien vascular plant species.

### Native species first record data

A total of 40,484,764 insect and 121,456,805 vascular plant records until year 2000 were sourced from GBIF (GBIF.org, 2020a,b). First, we removed records of fossil specimen. We also removed records of species listed in the Alien Species First Records database or in the Global Register of Introduced and Invasive Species (Pagad et al., 2018, accessed August 2020) to consider only native species records. We renamed synonym species using the R package *taxizedb* (Chamberlain & Arendsee, 2020) with the Integrated Taxonomic Information System. Then, for each species, the first record per country was extracted. This resulted in a total of 512,736 first records of 273,090 native insect species (~27% of known insect species) and 541,139 first records of 262,121 native vascular plant species (~80% of known vascular plant species) between 1750 and 2000.

### Model description and simulations

The null model is based on a statistical model described by Solow and Costello (2004), which estimates the rate of introduction of alien species from the discovery records. It defines the random variable *Yt* as the number of alien species first records in year *t*. *Y*t has a Poisson distribution with mean

$$\lambda_{t}=\sum_{s=1}^{t}\mu_{s}p_{\mathrm{st}}$$

where *µs* is the number of alien species introduced in year *s*, and *pst* is the probability that a species introduced in year *s* is discovered in year *t*. This probability is given by

$$p_{\mathrm{st}}=\pi_{t}\prod_{j=s}^{t‐1}\left(1‐\pi_{j}\right)$$

where *πt* is the probability of observing a species in year *t*, which is the sampling effort in year *t*.

The model requires three inputs: A model describing the introduction rate, the annual sampling effort (i.e. the probability of observing a species for each year) and the observed alien species first record rate. To build the null model, the parameter for the introduction rate was kept constant over time (*µt* = *µ*). Contrary to previous studies where the probability of observation was estimated in the model (Costello et al., 2007; Solow & Costello, 2004), we assumed that this value corresponds to the sampling effort, and used the rate of native species first records from GBIF as a proxy. The probability of observing a species in year *t* is, therefore, the proportion of native species first records in year *t*, given by

$$\pi_{t}=\frac{\beta_{t}}{\beta_{\text{Total}}}$$

where *βt* is the number of native species first records in year *t*, and *β*Total is the total number of native species first records on the whole period considered. We used native species first records—rather than all native species records—as a proxy for sampling effort because it can directly be compared with alien species first records because these two variables measure similar quantities: the number of new species—either alien or native—recorded each year in each country. Native species first record rate can thus be used to predict the expected number of alien species first records in each year, assuming that the probability of recording a new species is independent of whether it is native or alien (Belmaker et al., 2009)⁠.

The model was implemented with JAGS, using the *rjags* package (Plummer, 2019) in R v.4.0.4 (R Core Team, 2021), which is a program for analysis of Bayesian models using Markov Chain Monte Carlo simulation. For insects and vascular plants, a null model was fitted to the observed alien species first record rate. A uniform distribution was specified as a prior for the introduction rate parameter (*µ*). For each model, the value of *µ* was estimated with a 7,000 iterations Gibbs sampling with three chains. The first 4,000 iterations were discarded as burn‐in. Chain convergence was verified with Gelman and Rubin's convergence diagnostic (Gelman & Rubin, 1992)⁠. This procedure allowed to estimate the value of *µ* and to simulate 9,000 *λt* values (3,000 iterations*3 chains) per year *t* for insects and vascular plants. For each *λt*, a value was sampled in a Poisson distribution of mean *λt*, to obtain 9,000 simulated numbers of alien species first records per year *t*. Finally, 9,000 residuals (i.e. difference between observed and simulated number of alien species first records) were computed for each year.

### Linking invasion dynamics with world trade openness

World trade openness is a widely used economic index of the level of trade globalisation (but see Fujii, 2019 for a critical review)⁠. It measures the share of what is traded internationally compared with the overall market value of all final goods and services produced worldwide, and thus represents the importance of trade in the world economy. It is, therefore, an appropriate indicator to test whether broad variations in invasion rates follow large‐scale variations in globalisation. Our aim was not to evaluate the relative contribution of different socio‐economic aspects of globalisation at the scale of individual countries, as this question has been addressed in the recent literature (Amano et al., 2016; Chapman et al., 2017; Dawson et al., 2017), but to use a single, widely applied, index of globalisation that can be calculated for the past centuries⁠.

Trade openness was computed as the sum of all country imports and exports divided by the sum of all country GDP for each year from 1827 to 2000. Annual trade and GDP data were extracted from the TRADHIST database (Fouquin & Hugot, 2016)⁠. To test the effect of the annual variations of trade openness on invasion dynamics, we first smoothed trade openness with a cubic spline (smoothing parameter = 0.7) to be able to compute the derivative of trade openness.

### Statistical tests

We used Pearson's product moment correlation coefficient to measure the correlation between time series (*cor.test* function in R). First, we tested the link between simulated (mean of 9,000 simulations from the null model) and observed first record rates for insects and vascular plants. We then measured the correlation between the null model residuals (mean of 9,000 residuals per year) of the two taxonomic groups. Finally, we tested the correlation of the residuals with world trade openness derivative for each taxonomic group.

## RESULTS

### Importance of sampling effort

**FIGURE 1:**
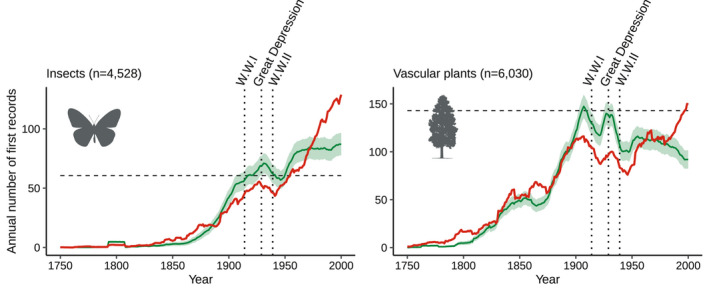
Simulated (green) and observed (red) alien species first record rate. For each taxonomic group, the simulated number of first records was computed with a null model which assumes a constant introduction rate (horizontal black dashed line) and accounts for temporal variation in sampling effort. For each taxon, simulated means and standard deviations are based on 9,000 simulations. To ease visualisation, simulated and observed data are here represented with a 15‐year moving average. Vertical black dotted lines represent dates of important events: The First World War (1914), Great Depression (1929) and Second World War (1939)

Most of the variation in the observed alien insect and vascular plant species first record rates can be explained by variations in sampling effort, as indicated by a strong correlation between simulated and observed first record rates (Figure 1; Pearson's *r* = 0.94 and 0.93 for insects and plants respectively; *p*‐value <0.001 for both groups).

### Estimated invasion dynamics

**FIGURE 2:**
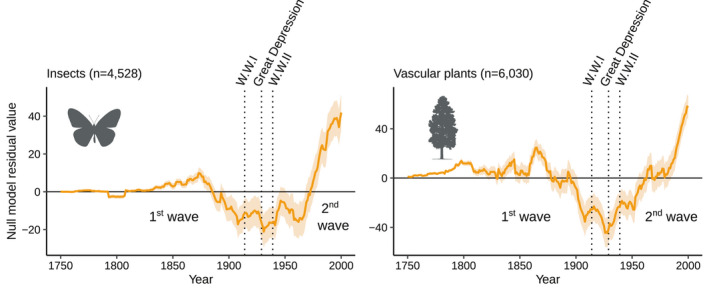
Variations in biological invasion rate unexplained by the null model. Mean and standard deviation of 9,000 residual values by year from the null model (i.e. difference between the simulated and observed alien species first records; Figure 1). Positive and negative values indicate that the observed number of alien species first records is higher and lower (respectively) than expected by the null model, thus reflecting the variations of invasion rate overtime. To ease visualisation, residual values are represented with a 15‐year moving average. Vertical black dotted lines represent dates of important events: The First World War (1914), Great Depression (1929) and Second World War (1939)

Insects and plants had similar estimated invasion dynamics (Pearson's *r* = 0.81, *p*‐value <0.001), with important fluctuations over the last centuries following a two‐wave pattern (Figure 2). The first wave of insect started in the 19th century as the observed first record rate between 1820 and 1870 was on average 143% higher than expected by the null model. For plants, the first wave of invasions was more spread out over time, as the rate of invasion was already important in the late 18th century. From 1750 to 1870, the observed alien plant first record rate was on average 39% higher than expected by the null model. The second wave of invasions started in the second half of the 20th century for both groups. The observed number of first records between 1970 and 2000 was 28% higher for insects and 22% higher for plants than expected by the null model. For both insects and plants, the two invasion waves were separated by a period of reduced invasion rates, roughly from 1900 to 1960, during which the observed first record rate decreased of 19% for insects and 20% for plants compared with the null model expectations. During that period, the rate of insect invasions remained relatively stable, whereas the rate of plant invasions resumed an upward trend after the Great Depression.

### Linking invasion dynamics with world trade openness

**FIGURE 3:**
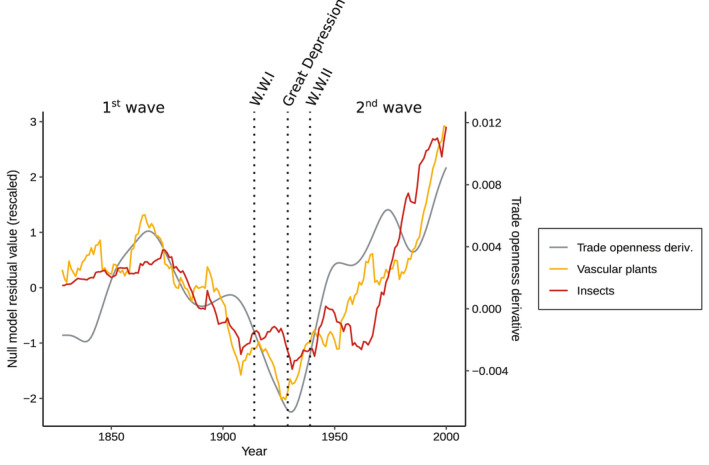
Correlation between trade openness variations and biological invasion dynamics. Coloured lines represent the mean of null model residuals for vascular plants and insects from years 1828 to 2000. The grey line represents world trade openness derivative, that is, the derivative of ((exports+imports)/world gross domestic product). Null model residual values were scaled to a mean of 0 and a variance of 1 for visualisation purposes and are represented with a 15‐year moving average. Dates of important events are indicated by the vertical black dotted lines: The First World War (1914), Great Depression (1929) and Second World War (1939)

The invasion dynamics of insects and plants were strongly correlated to world trade openness derivative (Figure 3; Pearson's *r* = 0.65 and 0.75 for insects and plants respectively; *p*‐value <0.001 for both groups). Trade openness derivative explained both the increases and decreases of the rate of invasions over the past centuries (Figure 3) and was the best economic predictor of biological invasion dynamics (Table S1).

## DISCUSSION

The rate of insect and vascular plant invasion did not rise continuously over the past two centuries. Instead, we found that, once temporal variations in sampling effort are accounted for, the rate of invasions fluctuated over time and followed two distinct waves of invasions. These two invasion waves were linked to large‐scale variations in world trade openness, which suggests that biological invasions accelerate when the economy becomes more globalised. Trade openness increased strongly between 1820 and 1870, marking the beginning of the first wave of globalisation, which was characterised by the expansion of the railroad network and the development of steamships (Baldwin & Martin, 1999; Federico & Tena‐Junguito, 2017)⁠. This first wave of globalisation has led to an acceleration of insect and plant invasions during that period. However, when trade openness ceased accelerating between 1870 and 1914, plummeting after the outbreak of the First World War, the rate of new invasions decreased rapidly. The rate of plant invasions started to increase again after the Great Depression, while insect invasion rate stayed relatively low until the second half of the 20th century. For both groups, the rate of invasions exploded in the 1970s, after the world recovered from the Great Depression and the Second World War, reaching unprecedented levels of trade openness by the end of the 20th century. Hence, variations in trade openness explains both the increasing number of invasions during the two waves of globalisation and the lower invasion rate during the first half of the 20th century. Although plant and insect invasion rates followed large‐scale variations in trade openness, the correlation is not perfect which suggests that other factors affected the spread of alien species at a global scale.

Interestingly, plant invasions had already taken off in the late 18th century, before the intensification of the first modern globalisation wave. This is probably a consequence of the numerous intentional introductions of alien plant species for ornamental purposes and food production that were already occurring at that time (van Kleunen et al., 2018)⁠. In addition, because plants can be transported as seeds (McNeill et al., 2011; Wilson et al., 2016)⁠, they may survive long journeys and depend less on fast transportation. By contrast, insects likely benefited from the advent of accelerated transportation in the 19th century to spread worldwide (Gippet et al., 2019), which could explain why the first invasion wave is more pronounced for insects than plants. But in contrast to plants, few records were available for both native and alien insect species before 1820; hence, no clear conclusion can be reached regarding insect invasions dynamics during that early period.

Our results demonstrate that the spread dynamics of insects and vascular plants—which represent the vast majority of described species (Scheffers et al., 2012)⁠ as well as the majority of known alien species (Seebens et al., 2017)—were linked to the two waves of globalisation. It is likely that other unintentionally introduced taxa, with similar introduction pathways as insects and plants, also follow the two waves of globalisation. However, dynamics may be different for intentionally introduced taxa, such as vertebrates, as their spread mostly depends on other pathways, like for example escape from zoos, farms or pet trade (Gippet & Bertelsmeier, 2021)⁠, introduction for biological control or through acclimatisation societies (Hulme et al. 2008)⁠, which are not directly related to globalisation dynamics.

Our findings also show that observed invasion dynamics are highly dependent on variations in sampling effort and, thus, highlight the importance of addressing this bias before reaching conclusions about temporal trends in global species accumulations. Even though we found that native species first records from GBIF could serve as proxy for sampling effort, this approach has some limitations. In particular, this proxy may underestimate sampling effort in the recent years because the pool of species remaining to be discovered decreases as new native species are recorded. As this pool reduces, the rate of native species first records inevitably tends to slow down even if sampling effort remains important. However, this is unlikely to have an impact for insects as only about 20% of all insect species have been described so far (Stork, 2018)⁠, which suggests that our proxy for insect sampling effort is appropriate and our results are robust for this group. For vascular plants, it is estimated that the majority of species have already been discovered (Joppa et al., 2011)⁠, which suggests that our approach could underestimate sampling effort for this taxonomic group in the more recent years. Consequently, the second wave of plant invasions may be less important than suggested by our results. But this issue would only affect the amplitude of the estimated second wave of invasions and not our conclusion concerning the global pattern of two waves of invasions.

Another limitation of our approach is that the invasion rate estimated from the null model does not reflect the exact rate of introductions because there is a time lag between the introduction of an alien species and the date of first observation (Aikio et al., 2010; Spear et al., 2021). However, estimating this time lag is very complex. It is likely to vary across taxa, as some species are better known or easier to detect than others. It might also change over time, as scientific knowledge and activity increase. Overall, such time lags may have been longer in the 19th century than in the more recent years, meaning that the first wave of invasions could have started earlier than estimated by our model. But overall, although this time lag could induce a temporal shift of the onset of the first invasion wave estimated in our analysis, it is unlikely to change the global dynamics of the two waves of invasions.

Our analysis show that biological invasions are strongly associated with temporal variations in world trade openness over the past two centuries, which we used as indicator of globalisation dynamics. Although the two globalisation waves consisted of a strong increase of international exchanges, their underlying trade flows strongly differed (Baldwin & Martin, 1999)⁠. The first globalisation wave was characterised by the hegemony of Great Britain and trade between the European countries and their colonies. The second wave was defined by the opening and dominance of the United States economy and the growing importance of emerging economies in global trade (Baldwin & Martin, 1999)⁠. Future research could explore in greater detail which countries were invaded and how alien species flows changed during the different phases of globalisation. This will be key to identifying the precise drivers of species introductions worldwide and will allow better predicting future invasions. This is especially important given that the future of globalisation, and consequently of biological invasions, remains highly uncertain. World trade openness has been decreasing since the 2007 financial crisis (Livesey, 2018; Witt, 2019) and the COVID‐19 pandemic also slowed down international exchanges in 2020 (Enderwick & Buckley, 2020; Vidya & Prabheesh, 2020; WTO, 2020)⁠, potentially decreasing rates of new invasions as it happened after the first wave of globalisation.

Overall, our findings contest the widespread idea of an inexorable acceleration of biological invasions over the last centuries. Instead, we show that the world experienced two waves of invasions since the Industrial Revolution, which were associated with the two waves of globalisation. Even though it has long been recognised that human activity is responsible for the vast majority of biological invasions, most research has focussed on the role of habitat or species characteristics affecting invasion success, rather than on human‐mediated dispersal (Catford et al., 2009; Ricciardi et al., 2017)⁠. Our findings emphasise the urgency to get a better understanding of how globalisation affects the accidental transport of invasive species because biological theory alone cannot explain current invasions, or predict those likely to happen in the future (Kueffer, 2017)⁠.

## AUTHORSHIP

A.B., J.M.W.G. and C.B. designed the study. A.B. performed the research. A.B., J.M.W.G. and C.B. all contributed to the writing of the paper.

### PEER REVIEW

The peer review history for this article is available at https://publons.com/publon/10.1111/ele.13863.

## Supporting information

Supplementary Material

## Data Availability

Data and code used to perform the analysis and generate the figures are available at: https://doi.org/10.5281/zenodo.5153707.
