# Supplementary material for: Insect and plant invasions follow two waves of globalisation
Source: Ecol Lett. 2021 Aug 22;24(11):2418–26. doi: 10.1111/ele.13863 (PMC9290749; doi:10.1111/ele.13863)
Supplement: Supplementary file 1 — Supplementary Material [file ELE-24-2418-s001.docx]

**Supplementary Material S1**

To test which economic index was the best predictor of insect and plant invasion dynamics when sampling bias is accounted for, we included different variables in the model: trade openness, world trade value (log-transformed), world GDP (log-transformed) and the derivative of each of these three indexes (i.e., variation of the index from one year to the other). Before computing the derivatives, each variable was smoothed with a cubic spline (smoothing parameter = 0.7). Derivatives were then rescaled between 0 and 1 to avoid negative values. Each variable was computed from 1828 to 2000 and the models were fitted over this period. We used the same model as described in the main text, but contrary to the null model, where the introduction rate is constant overtime, we here defined a linear relationship between the introduction rate and the economic index. The number of introductions in year *t* is thus given by a*x*t*, where x*t* is the value of the economic index in year *t*. We also fitted a null model (i.e., with constant introduction rate) from 1828 to 2000 to compare the results with the “complete” models and thus test if biological invasions are dependent of the economic indexes. The simulations were performed following the same methodology as described in the main text. We then computed the root-mean-square error (RMSE) for each model. The results are presented in Table S1.

Table S1: Model RMSE comparison for each economic index and for the null model.

|  | **trade openness derivative** | trade openness | log(trade) | log(GDP) | log(trade) derivative | log(GDP) derivative | null model |
| --- | --- | --- | --- | --- | --- | --- | --- |
| Insects | **16.2** | 17.7 | 17.6 | 17.6 | 17.7 | 23.6 | 18.4 |
| Vascular plants | **40.9** | 45.3 | 43 | 42.9 | 44.7 | 58.2 | 42.6 |

For both insects and plants, trade openness derivative is the best economic predictor of biological invasion dynamics over the last centuries. Including trade openness derivative in the model also improved the fit compared to the null model. This confirms the link between globalization and biological invasions dynamics, and that the rate of invasion did not continuously increase but fluctuated following the two modern globalization waves.

One limitation of this approach is that the model starts in 1828 since the economic variables are available from that date onwards. This can be a problem for plants as many were introduced before 1828, we thus loose information, affecting the fit of the model (null model RMSE = 32.4 and 42.6 for vascular plants when the null model starts in 1750 and 1828, respectively)
